# Meteorological Table, for the Month of June, 1853

**Published:** 1853-07

**Authors:** Lawrence Young

**Affiliations:** Near Louisville, Ky.


### METEOROLOGICAL TABLE,

*For the Month of June,* 1853.

BY LAWRENCE YOUNG, ESQ., NEAR LOUISVILLE, KY.

| Day of month. | Morning. | 2 o'clock, P. M. | Evening. | Mean Temperature. | Barometer. | Rain-guage. | Course of Wind. | Remarks. |
|---|---|---|---|---|---|---|---|---|
| 1 | 50 | 89 | 77 | 72 | 29.47 | | S. W. | Clear. |
| 2 | 67 | 83 | 78 | 76 | 29.54 | | S. S. W. | Cloudy. |
| 3 | 69 | 86 | 74 | 77 | 29.57 | | W. S. W. | Variable. |
| 4 | 65 | 90 | 78 | 78 | 39.62 | | S. W. | Do. |
| 5 | 64 | 82 | 78 | 75 | 29.58 | | W. | Clear. |
| 6 | 69 | 86 | 74 | 77 | 29.51 | | S. S. W. | Variable. |
| 7 | 60 | 77 | 66 | 68 | 29.49 | 18 | N. N. W. | Do. |
| 8 | 44 | 78 | 67 | 65 | 29.67 | | S. E. | Clear. |
| 9 | 50 | 87 | 73 | 70 | 29.53 | | W. | Variable. |
| 10 | 57 | 90 | 77 | 75 | 29.56 | | W. S. W. | Clear. |
| 11 | 60 | 91 | 77 | 76 | 29.60 | | E. N. E. | Do. |
| 12 | 61 | 91 | 80 | 77 | 29.68 | | E. | Variable. |
| 13 | 63 | 93 | 76 | 77 | 29.71 | | W. S. W. | Clear. |
| 14 | 62 | 93 | 73 | 76 | 29.70 | 56 | W. | Variable. |
| 15 | 63 | 81 | 75 | 73 | 29.60 | | N. | Cloudy. |
| 16 | 64 | 91 | 78 | 78 | 29.45 | | W. | Variable. |
| 17 | 63 | 86 | 74 | 74 | 29.48 | | W. S. W. | Clear. |
| 18 | 55 | 86 | 74 | 72 | 29.52 | | W. | Do. |
| 19 | 55 | 92 | 78 | 75 | 29.51 | | S. S. W. | Do. |
| 20 | 61 | 96 | 81 | 79 | 29.54 | | S. S. W. | Variable. |
| 21 | 71 | 93 | 78 | 80 | 29.50 | | S. | Do. |
| 22 | 71 | 91 | 82 | 81 | 29.51 | | S. S. W. | Do. |
| 23 | 76 | 75 | 68 | 73 | 29.37 | | W. S. W. | Do. |
| 24 | 60 | 76 | 68 | 68 | 29.70 | | W. S. W. | Do. |
| 25 | 56 | 77 | 69 | 67 | 29.67 | | N. | Do. |
| 26 | 60 | 88 | 78 | 75 | 29.65 | | E. | Do. |
| 27 | 68 | 91 | 78 | 79 | 29.53 | | W. S. W. | Do. |
| 28 | 61 | 96 | 82 | 80 | 29.56 | | W. S. W. | Do. |
| 29 | 68 | 97 | 84 | 83 | 29.51 | | W. N. W. | Do. |
| 30 | 66 | 94 | 83 | 81 | 29.55 | | N. W. | Do. |
| | | | | 75¼ | | 74 | | |

